# Roles of Aminoacyl-tRNA Synthetases in Cancer

**DOI:** 10.3389/fcell.2020.599765

**Published:** 2020-11-27

**Authors:** Zheng Zhou, Bao Sun, Anzheng Nie, Dongsheng Yu, Meng Bian

**Affiliations:** ^1^Department of Chinese Medicine, The First Affiliated Hospital of Zhengzhou University, Zhengzhou, China; ^2^Department of Pharmacy, The Second Xiangya Hospital, Central South University, Changsha, China; ^3^Institution of Clinical Pharmacy, Central South University, Changsha, China

**Keywords:** aminoacyl-tRNA synthetase, ARS-interacting multifunctional protein, cancer, pathogenesis, therapeutics

## Abstract

Aminoacyl-tRNA synthetases (ARSs) catalyze the ligation of amino acids to their cognate transfer RNAs (tRNAs), thus playing an important role in protein synthesis. In eukaryotic cells, these enzymes exist in free form or in the form of multi-tRNA synthetase complex (MSC). The latter contains nine cytoplasmic ARSs and three ARS-interacting multifunctional proteins (AIMPs). Normally, ARSs and AIMPs are regarded as housekeeping molecules without additional functions. However, a growing number of studies indicate that ARSs are involved in a variety of physiological and pathological processes, especially tumorigenesis. Here, we introduce the roles of ARSs and AIMPs in certain cancers, such as colon cancer, lung cancer, breast cancer, gastric cancer and pancreatic cancer. Furthermore, we particularly focus on their potential clinical applications in cancer, aiming at providing new insights into the pathogenesis and treatment of cancer.

## Introduction

The genetic information in the organism is transformed into functional proteins through transcription and translation. Of these, aminoacyl-tRNA synthetases (ARSs) catalyze the aminoacylation of transfer RNAs (tRNAs), thereby playing an important role in translation (Kwon et al., 2019). In this process, each ARS interacts with an amino acid and an ATP molecule to produce a high-energy aminoacyl adenylate (aa-AMP) intermediate and a pyrophosphate (PPi) molecule (Kwon et al., 2019). Subsequently, the intermediate binds to the cognate tRNA and transfers the amino acid to it. Eventually, the aminoacylated tRNA is recruited to the ribosome to participate in protein synthesis. Actually, there are thirty-six ARSs in a typical human cell, and they can function in the cytoplasm or mitochondria (Antonellis and Green, 2008). Of note, in addition to being in free form in the cytoplasm, nine ARSs interact with three ARS-interacting multifunctional proteins (AIMPs) to form a multi-tRNA synthetase complex (MSC) (Hyeon et al., 2019). Among them, AIMPs mainly play scaffolding roles in the assembly of the MSC. Furthermore, ARSs are usually divided into two categories based on their structural characteristics (Pang et al., 2014). The first is characterized by a Rossman fold in its catalytic domain, whereas the second has three conserved sequence motifs, namely Motif 1, Motif 2 and Motif 3 (Pang et al., 2014).

Generally, ARSs and AIMPs are considered as housekeeping molecules. However, increasing evidence indicates that these molecules are involved in various physiological and pathological processes, including cysteine polysulfidation, angiogenesis, posttranslational modification, immune response and nervous system development (Akaike et al., 2017; Fu et al., 2017; He et al., 2018; Mullen et al., 2020; Zhu et al., 2020). Strikingly, ARSs and AIMPs are closely related to tumorigenesis (Yin et al., 2016; Gao et al., 2019; Zhou Z. et al., 2020). Certain ARSs, such as isoleucyl-tRNA synthetase 2 (IARS2), lysyl-tRNA synthetase (KRS) and asparaginyl-tRNA synthetase (NRS), have been shown to promote cancer development (Kim et al., 2012; Fang et al., 2018; Yeom et al., 2020), while AIMPs and seryl-tRNA synthetase (SerRS) usually exert anti-tumor effects (Li Y. et al., 2019; Zhou Z. et al., 2020). Furthermore, some ARSs are abnormally expressed in various cancer tissues and have diagnostic and prognostic value (Jung et al., 2017; Lee and Kim, 2019). For instance, methionyl-tRNA synthetase (MRS) was highly expressed in non-small cell lung cancer (NSCLC) cells that had metastasized to lymph nodes but was weakly expressed in cells adjacent to the lesion (Lee and Kim, 2019). Importantly, MRS/CD45 dual immunofluorescent staining increased the diagnostic rate of lymph node metastasis in NSCLC, indicating that MRS might serve as a potential biomarker for cancer metastasis (Lee and Kim, 2019). In this review, we not only focus on the roles of ARSs and AIMPs in the development of cancer, but also emphasize the value of these molecules in the diagnosis and treatment of cancer.

## Roles of ARSs in Cancer

**FIGURE 1:**
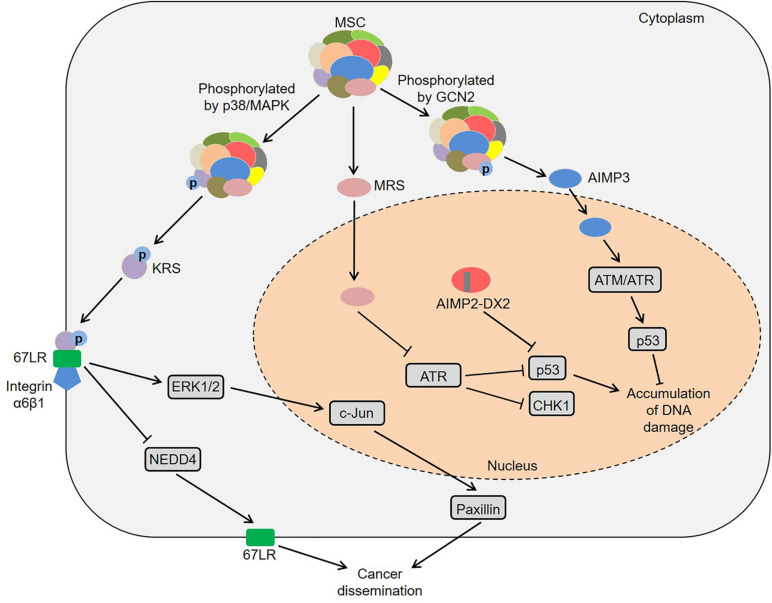
Common pathways mediated by ARSs in cancer development. KRS is phosphorylated by p38/MAPK and then dissociates from the MSC. Subsequently, the KRS interacts with 67LR that is localized in the cell membrane, which prevents NEDD4 from ubiquitinating 67LR and enhances laminin-induced cell migration. Moreover, KRS forms a complex with 67LR and integrin α6β1 to mediate ERKs/c-Jun activation and paxillin expression. MRS increases the lysine homocysteinylation of ATR, thereby inhibiting ATR and its downstream CHK1 and p53. AIMP2-DX2 reduces the pro-apoptotic activity of AIMP2 by competitively binding to p53 with AIMP2. Furthermore, MRS is phosphorylated by GCN2, resulting in a conformational change of MRS and subsequent dissociation of AIMP3 from the MSC. The released AIMP3 interacts with ATM/ATR to up-regulate the expression of p53, thereby responding to DNA damage. ARSs, aminoacyl-tRNA synthetases; AIMPs, ARS-interacting multifunctional proteins; KRS, lysyl-tRNA synthetase; MAPK, mitogen-activated protein kinase; MSC, multi-tRNA synthetase complex; 67LR, 67-kDa laminin receptor; NEDD4, neural precursor cell expressed developmentally downregulated 4; ERKs, extracellular signal-regulated kinases; MRS, methionyl-tRNA synthetase; ATR, ataxia telangiectasia and Rad3-related protein; CHK1, checkpoint kinase-1; AIMP2-DX2, AIMP2 lacking exon 2; GCN2, general control non-repressed-2; ATM, ataxia telangiectasia-mutated.

Besides the catalytic domains, ARSs also contain other domains, including leucine zipper, glutathione *S*-transferase, WHEP and endothelial monocyte activating polypeptide II domains, which seem to provide the possibility for the functional diversity of ARSs (Kwon et al., 2019). In fact, their abnormal expression, cellular localization and molecular interactions cause a variety of human diseases (Park et al., 2008; D’Hulst et al., 2020; Wu et al., 2020). Among them, some ARS-mediated signaling pathways are involved in the regulation of multiple tumors (Figure 1). Laminin activated p38/mitogen-activated protein kinase (MAPK) by binding to integrin, which induced the phosphorylation of KRS at the T52 residue in A549 cells (Kim et al., 2012). Subsequently, the phosphorylated KRS dissociated from the MSC and interacted with 67-kDa laminin receptor (67LR) that was localized in the cell membrane. This interaction prevented neural precursor cell expressed developmentally downregulated 4 (NEDD4) from ubiquitinating 67LR as well as enhanced laminin-induced cell migration (Kim et al., 2012). Analogously, Nam et al. found that KRS played a vital role in the invasive dissemination of colon cancer spheroids in 3D collagen I gels (Nam et al., 2015). Membranous KRS formed a complex with 67LR and integrin α6β1 to mediate extracellular signal-regulated kinase 1/2 (ERK1/2)/c-Jun activation and paxillin expression in colon cancer cells, thereby promoting cancer dissemination (Nam et al., 2015). These findings indicate that KRS plays an important role in cancer metastasis.

Moreover, certain ARSs and AIMPs could regulate tumorigenesis by p53 (Park et al., 2005; Choi et al., 2011; Wang et al., 2018). High-fat diet induced colonic lysine homocysteinylation through MRS, which led to the accumulation of DNA damage and colorectal cancer-like phenotypes in the colon of mice and rats (Wang et al., 2018). Specifically, MRS increased the homocysteinylation of ataxia telangiectasia and Rad3-related protein (ATR) at five lysine residues by binding to ATR, thereby inhibiting ATR and its downstream moleculars checkpoint kinase-1 (CHK1) and p53 (Wang et al., 2018). Conversely, MRS inhibitors, *N*-acetyl cysteine and acetylhomocysteine thioether, could reverse tumor-like phenotypes in both HCT116 cells and rats, which provided new ideas for understanding the mechanism of high-fat diet-associated colorectal cancer (Wang et al., 2018). Considering that amino acids are the substrates of ARSs, high-protein diets or ketogenic diets may participate in tumor development by regulating ARSs, which deserves more in-depth research. Interestingly, a splicing variant of AIMP2 lacking exon 2 (AIMP2-DX2) was highly expressed in human lung cancer tissues, and the ratio of AIMP2-DX2 to AIMP2 was negatively correlated with patient survival (Choi et al., 2011). Further studies found that this splicing variant reduced the pro-apoptotic activity of AIMP2 by competitively binding to p53 with AIMP2 (Choi et al., 2011). Of note, AIMP3 heterozygous mice exhibited spontaneous tumorigenesis in multiple organs (Park et al., 2005). AIMP3 directly interacted with ataxia telangiectasia-mutated (ATM)/ATR to up-regulate the expression of p53, thereby responding to DNA damage (Park et al., 2005). Under UV stimulation, the Ser662 site of MRS was phosphorylated by general control non-repressed-2 (GCN2), resulting in a conformational change of MRS and subsequent dissociation of AIMP3 from the MSC (Kwon et al., 2011). The released AIMP3 was translocated into the nucleus and participated in DNA repair (Kwon et al., 2011). Additionally, ARSs also contribute to the development of specific tumors via other pathways, such as colon cancer, lung cancer, breast cancer and pancreatic cancer (Shin et al., 2008; Katsyv et al., 2016; Jeong et al., 2018; Nam et al., 2018).

### ARSs and Colon Cancer

Colon cancer is one of the most common gastrointestinal cancers and has the second and third highest mortality rates among male and female patients, respectively (Bin et al., 2020). Interestingly, KRS was involved in colon cancer metastasis by inducing M2 macrophage polarization (Nam et al., 2018). In this process, KRS dissociated from the MSC after S207 phosphorylation and then translocated to the nucleus (Nam et al., 2018). The nuclear KRS induced growth arrest-specific 6 (GAS6) transcription by activating microphthalmia-associated transcription factor (MiTF), which promoted the M2 polarization of neighboring macrophages (Nam et al., 2018). Subsequently, M2 macrophages secreted soluble factors, such as growth-regulated oncogene-α (GROα), fibroblast growth factor 2 (FGF2) and macrophage colony-stimulating factor (M-CSF). These factors not only induced the activation of intracellular signals in cancer cells, but also activated the adjacent cancer-associated fibroblasts and promoted the secretion of laminins, which ultimately remodeled the tumor microenvironment and exacerbated cancer metastasis (Nam et al., 2018). Furthermore, caspase-8 could cleave KRS and expose the PDZ binding motif located at the C-terminus of KRS in HCT116 cells (Kim S.B. et al., 2017). The exposed PDZ binding motif interacted with syntenin, facilitating the dissociation of KRS from the MSC and the subsequent exosomic secretion of KRS from colorectal carcinoma cells. Importantly, the KRS-containing exosomes induced the M1 polarization and migration of macrophages, indicating that KRS might be involved in cancer-induced inflammation (Kim S.B. et al., 2017). These results suggest that KRS is closely associated with the development of colon cancer.

**FIGURE 2:**
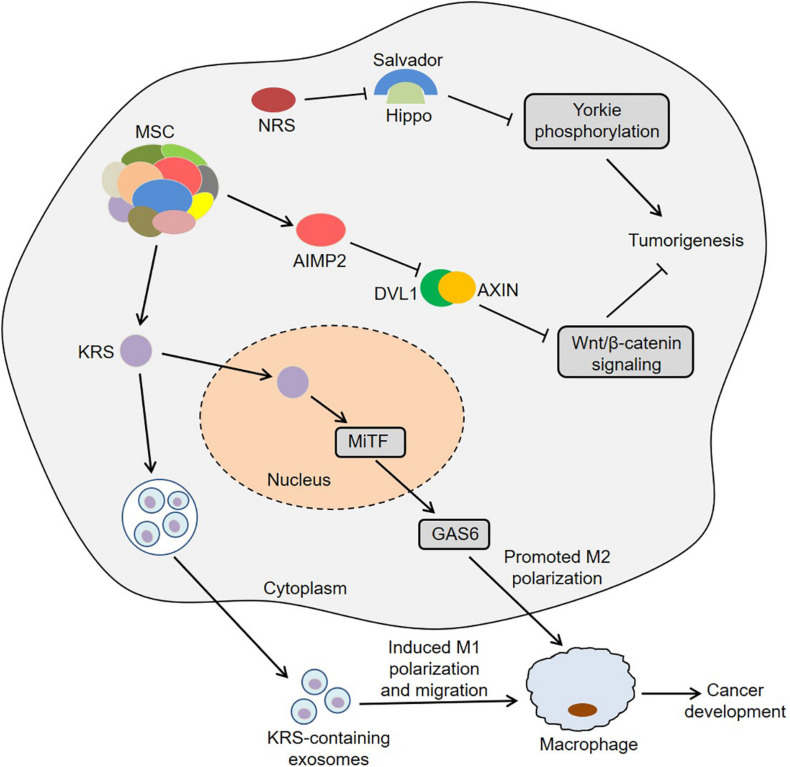
Roles of ARSs and AIMPs in the biology of colon cancer. KRS translocates to the nucleus and then induces GAS6 transcription by activating MiTF, which promotes the M2 polarization of neighboring macrophages and then facilitates cancer metastasis. KRS-containing exosomes are released by cancer cells and then induce M1 polarization and migration of macrophages. NRS blocks the interaction between Salvador and Hippo by binding to Salvador, thereby activating Yorkie target genes via decreasing Yorkie phosphorylation. AIMP2 blocks the interaction between DVL1 and AXIN and thus inhibits the activity of Wnt/β-catenin signaling. ARSs, aminoacyl-tRNA synthetases; AIMPs, ARS-interacting multifunctional proteins; KRS, lysyl-tRNA synthetase; MiTF, microphthalmia-associated transcription factor; GAS6, growth arrest-specific 6; NRS, asparaginyl-tRNA synthetase; DVL1, disheveled-1; AXIN, axis inhibition protein.

Zhong et al. (2015) observed that IARS2 was highly expressed in human colon cancer tissues, and knockdown of IARS2 could inhibit RKO cell proliferation, suggesting that IARS2 might trigger the development of colon cancer. Furthermore, NRS induced Yorkie-mediated tumor phenotypes in a *Drosophila* model (Yeom et al., 2020). During this process, NRS blocked the interaction between Salvador and Hippo by binding to Salvador, thereby activating Yorkie target genes via decreasing Yorkie phosphorylation (Yeom et al., 2020). Notably, YAP, a mammalian homolog of Yorkie, target genes were upregulated in colon cancer C26 cells, and NRS inhibitor TirB decreased the levels of YAP target genes and suppressed cell proliferation in C26 cells, indicating that NRS might regulate the development of colon cancer by Hippo signaling pathway (Yeom et al., 2020). Intriguingly, the hemizygous deletion of AIMP2 promoted epithelial cell proliferation and intestinal stem cell expansion in the intestinal crypt, which resulted in the formation of intestinal adenoma in *Apc*^Min/+^ mice (Yum et al., 2016). Mechanistically, AIMP2 bound to Disheveled-1 (DVL1) and blocked the interaction between DVL1 and axis inhibition protein (AXIN), thus inhibiting the activity of Wnt/β-catenin signaling (Yum et al., 2016). In short, ARSs and AIMPs are closely related to the biology of colon cancer (Figure 2).

### ARSs and Lung Cancer

Lung cancer is the leading cause of cancer-related deaths worldwide, with dismal prognosis and poor clinical outcomes (Drula et al., 2020). Previous studies found that leucyl-tRNA synthetase (LRS) was significantly up-regulated in A549 lung cancer cells, and its mRNA was also highly expressed in primary lung cancer tissues (Shin et al., 2008). Importantly, the growth and migration of LRS knockdown A549 cells were inhibited, indicating that this molecule played an important role in the development of lung cancer (Shin et al., 2008). Furthermore, tyrosyl-tRNA synthetase (TyrRS) and microtubule-actin crosslinking factor 1 (MACF-1) were at higher abundance in lung adenocarcinoma tissues than adjacent normal tissues (Zhou et al., 2013). Among them, the levels of TyrRS were difference between N1, N2, and N3, while the levels of MACF-1 were associated with T stage, N stage and clinical stage. Meanwhile, patients expressing TyrRS or MACF-1 had a significantly increased risk of death (Zhou et al., 2013). Di et al. (2019) observed that the expression of IARS2 was higher in NSCLC tissues, and silencing IARS2 could inhibit the activity of A549 and H1299 cells and reduce the tumorigenicity of lung cancer cells in nude mice. Mechanistically, the phosphorylation levels of protein kinase B (AKT) and mammalian target of rapamycin (mTOR) were significantly lower in IARS2-silenced lung cancer cells, while AKT activator SC79 partially restored the AKT phosphorylation and lung cancer cell proliferation, suggesting that IARS2 might be involve in tumorigenesis through the AKT/mTOR pathway (Di et al., 2019).

**FIGURE 3:**
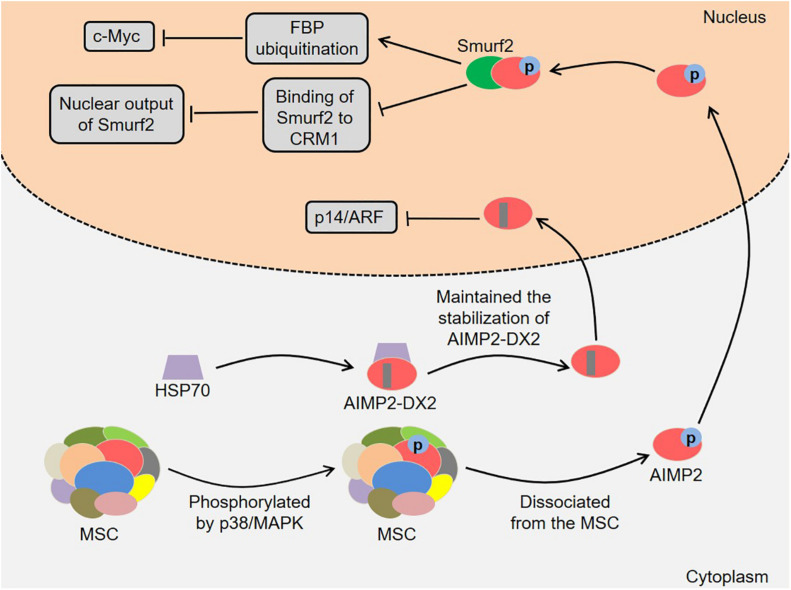
Roles of AIMP2 and AIMP2-DX2 in the development of lung cancer. AIMP2 dissociates from the MSC and translocates to the nucleus. The nuclear AIMP2 interacts with Smurf2, thereby enhancing FBP ubiquitination and down-regulating c-Myc. Meanwhile, the interaction also inhibits the binding of Smurf2 to CRM1, which enhances TGF-β signal by reducing the nuclear output of Smurf2. HSP70 inhibits the Siah1-dependent AIMP2-DX2 ubiquitination by interacting with AIMP2-DX2, thereby maintaining the stabilization of AIMP2-DX2. AIMP2-DX2 inhibits p14/ARF by directly binding to it, thereby suppressing oncogene-induced apoptosis and senescence. AIMP2, ARS-interacting multifunctional protein 2; AIMP2-DX2, AIMP2 lacking exon 2; MSC, multi-tRNA synthetase complex; MAPK, mitogen-activated protein kinase; Smurf2, Smad ubiquitin regulatory factors 2; FBP, FUSE-binding protein; CRM1, chromosomal region maintenance 1; TGF-β, transforming growth factor-β; HSP70, heat shock protein 70; Siah1, Seven *in absentia* homolog 1.

In addition, AIMP2 and its splicing variant are involved in the development of lung cancer (Figure 3). AIMP2 had multidirectional tumor-suppressive activity, which mediated the apoptotic response to DNA damage, tumor necrosis factor α (TNF-α)-induced cell death and sensitivity to anti-proliferative transforming growth factor-β (TGF-β) signal in a dose-dependent manner (Choi et al., 2009). Of note, heterozygous AIMP2 mice with low AIMP2 levels showed high susceptibility to lung and colon carcinogenesis (Choi et al., 2009). Kim et al. (2016) found that AIMP2 was phosphorylated by TGF-β-activated p38/MAPK at S156 in HeLa cells. Next, the phosphorylated AIMP2 dissociated from the MSC and translocated to the nucleus. The nuclear AIMP2 interacted with Smad ubiquitin regulatory factors 2 (Smurf2), thereby enhancing FUSE-binding protein (FBP) ubiquitination and down-regulating c-Myc (Kim et al., 2016). Meanwhile, the interaction between AIMP2 and Smurf2 also inhibited the binding of Smurf2 to chromosomal region maintenance 1 (CRM1), which enhanced TGF-β signal by reducing the nuclear output of Smurf2 (Kim et al., 2016). Notably, the A549 cells expressing AIMP2 S156A mutant were more likely to generate tumors *in vivo*, indicating that AIMP2 played an essential role in regulating the growth inhibitory activity of TGF-β (Kim et al., 2016). Lim et al. (2020) revealed that the levels of heat shock protein 70 (HSP70) and AIMP2-DX2 were positively correlated in lung cancer cells, and HSP70 could regulate AIMP2-DX2 level to some extent. Specifically, HSP70 inhibited the Seven *in absentia* homolog 1 (Siah1)-dependent AIMP2-DX2 ubiquitination by interacting with AIMP2-DX2, thereby maintaining the stabilization of AIMP2-DX2 (Lim et al., 2020). Meaningfully, a compound BC-DXI-495 blocked the interaction of HSP70 with AIMP2-DX2 and thus inhibited cancer progression *in vitro* and *in vivo* (Lim et al., 2020). Moreover, AIMP2-DX2 inhibited p14/ARF by directly binding to it, thereby suppressing oncogene-induced apoptosis and senescence (Oh et al., 2016). SLCB050 prevented their interaction and inhibited the development of lung cancer induced by ectopic expression of AIMP2-DX2 in mice, which provided a new idea for cancer treatment (Oh et al., 2016).

### ARSs and Breast Cancer

Breast cancer is the most common malignancy in women, which threatens the lives of patients and seriously affects their quality of life (Au et al., 2020). Recent studies discovered that four single nucleotide polymorphisms (SNPs) in ARS genes, including rs34087264 in alanyl-tRNA synthetase (AlaRS), rs801186 in histidyl-tRNA Synthetase (HARS), rs193466 in arginyl-tRNA synthetase (RARS) and rs2273802 in tryptophanyl-tRNA synthetase (WARS), were significantly associated with an increased risk of breast cancer in Chinese population (He et al., 2015). Accumulating evidence has implicated the insulin-like growth factor (IGF) signaling with breast cancer biology (Ireland et al., 2018; De Vincenzo et al., 2019). IGF overexpression significantly promoted cell proliferation and tumor formation in mice (Pacher et al., 2007). Notably, MRS was up-regulated in the MCF7 cells stably overexpressing IGF1 or IGF2, indicating that MRS might mediate the tumorigenesis regulated by IGF (Pacher et al., 2007). It is well known that the activity of cyclin-dependent kinase 4 (CDK4) is inhibited by p16^INK4a^ (Cen et al., 2012; Liao et al., 2020). Intriguingly, MRS maintained the stability of CDK4 by interacting with CDK4 and heat shock protein 90 (HSP90), thereby regulating the cell cycle (Kwon et al., 2018). Since MRS could compete with p16^INK4a^ for binding to CDK4, the stabilizing effect of MRS on CDK4 was more significant in p16^INK4a^-negative breast cancer cells (Kwon et al., 2018). Suppression of MRS reduced the tumorigenic ability of MDA-MB-231 cells *in vivo*, implying that MRS was involved in the development of breast cancer (Kwon et al., 2018). Furthermore, Qi et al. (2019) identified 9 differentially expressed-methylated hub genes in breast cancer. Of these, glutamyl-prolyl-tRNA synthetase (EPRS) was up-regulated and was related to poor clinical outcomes in breast cancer, suggesting that EPRS might be regulated by DNA methylation and contributed to the development of breast cancer (Qi et al., 2019). Similarly, EPRS was found to be highly expressed in estrogen receptor positive (ER+) breast cancer tissues and was associated with reduced overall survival in TCGA and METABRIC datasets (Katsyv et al., 2016). Further studies discovered that EPRS regulated cell cycle and estrogen response genes, indicating that EPRS might serve as an important regulator of cell proliferation and estrogen signaling in ER+ breast cancer (Katsyv et al., 2016). In addition, AIMP1 significantly inhibited the growth of breast cancer 4T1 cells in mice, accompanied by decreased myeloid-derived suppressor cell (MDSC) population in the spleens and tumor sites (Hong et al., 2016). Meanwhile, AIMP1 not only suppressed the expansion of MDSCs *in vitro*, but also reduced the expression of IL-6, NO and arginase-1 in MDSCs (Hong et al., 2016). In-depth studies found that AIMP1 inhibited the expansion and suppressive functions of MDSCs by reducing the activity of signal transducers and activators of transcription (STATs), AKT and ERK, indicating that AIMP1 might be a key target of breast cancer immunotherapy strategies (Hong et al., 2016). Strikingly, RARS overexpression significantly reduced AIMP1 secretion in HeLa and MCF7 cells, indicating that RARS might participate in tumorigenesis by regulating the secretion of AIMP1 in cancer cells (Bottoni et al., 2007).

### ARSs and Other Cancers

Gastric cancer is the fourth most common malignant tumor, characterized by high mortality, low rates of early diagnosis and poor prognosis (Shafabakhsh et al., 2020). KRS was highly expressed in gastric cancer tissues and tumor-associated inflammatory cells (Kim B.H. et al., 2014). Meaningfully, the high levels of KRS in tumor cells were associated with shorter overall survival, while its high levels in tumor-associated inflammatory cells were associated with longer overall survival, indicating that KRS might act as a potential biomarker for gastric cancer (Kim B.H. et al., 2014). Analogously, another research found that both WARS and indoleamine 2,3-dioxygenase 1 (IDO1) were highly expressed in Epstein-Barr virus-associated and microsatellite instability-high subtypes of gastric cancer (Lu et al., 2020). The expression of WARS and IDO1 was related to poor prognosis in p53-aberrant and p53-wildtype subtypes, while the expression of WARS was related to better prognosis in microsatellite instability subtype (Lu et al., 2020). Tian et al. (2017) demonstrated that four SNPs in cysteinyl-tRNA synthetase (CARS), including rs384490, rs7394702, rs2071101, and rs729662, were associated with gastric cancer risk. Of these, rs384490 and rs7394702 were found to alter the DNA methylation or transcription factor response elements of CARS by silico analysis, and rs384490 and rs729662 were found to regulate the expression of CARS-related genes by expression quantitative trait loci analysis, indicating that CARS might be involved in the development of gastric cancer (Tian et al., 2017). Moreover, TyrRS knockdown significantly inhibited the proliferation of gastric cancer cell lines AGS, HGC-27 and MGC-803, while TyrRS overexpression promoted the tumor growth in HGC-27 xenograft models (Zhang et al., 2020). Further studies found that TyrRS could facilitate gastric cancer progression and homologous recombination process by regulating phosphatidylinositol 3-kinase (PI3K)/AKT signaling (Zhang et al., 2020). Melanoma is a type of aggressive skin cancer with high heterogeneity (Pierce and Simmons, 2020). WARS could regulate the proliferation, migration and invasion of uveal melanoma cells via inducing the activation of PI3K/AKT/mTOR pathway, suggesting that WARS might be is a novel tumorigenic factor (Yang et al., 2020). Liang et al. (2017) observed that AIMP1 was essential for the bone marrow-derived dendritic cell (BMDC) vaccine-mediated protection against melanoma. In detail, AIMP1 enhanced the T-helper type 1 (T_H_1) polarization partly through p38/MAPK signaling pathway, thereby exerting an antitumor effect (Liang et al., 2017).

Furthermore, ARSs were also associated with other cancers, such as liver cancer, pancreatic cancer, leukemia and oral cancer (Chen et al., 2011; Paley et al., 2011; Ma et al., 2013; Lee et al., 2015). Previous studies found that WARS and fumarate hydratase were involved in the hepatitis B virus (HBV)-induced angiogenesis in rat primary hepatocytes and HepG2 cells by LC-MS/MS analysis, indicating that these two proteins might act as potential anti-angiogenic targets in liver cancer therapy (Zhang et al., 2009). Nuclear transcription factor nuclear factor of activated T cells 5 (NFAT5) has been implicated in the pathogenesis of various human cancers (Jiang et al., 2019; Liu et al., 2020). Interestingly, HBV enhanced liver cancer progression by suppressing NFAT5 (Qin et al., 2017). In this process, HBV not only inhibited NFAT5 expression by inducing the hypermethylation of NFAT5 promoter, but also inhibited NFAT5 through MAPK activated by miR-30e-5p (Qin et al., 2017). Subsequently, the low expression of NFAT5 could up-regulate aspartyl-tRNA synthetase 2 (DARS2), thereby promoting liver cancer development by accelerating cell cycle progression and inhibiting cell apoptosis. MUC1, a member of the mucin family, is closely associated with cancer pathology (Kufe, 2009). Knockdown of threonyl-tRNA synthetase (TRS) could reduce the level of MUC1 and thus inhibit the migration of pancreatic cancer cells (Jeong et al., 2018). Moreover, IARS2 knockdown increased the levels of p53 and p21 and decreased the levels of proliferating cell nuclear antigen (PCNA) and eukaryotic translation initiation factor 4E (eIF4E) in HL-60 cells, thereby inhibiting cell proliferation (Li H. et al., 2019). These results indicate that IARS2 may participate in the development of acute myeloid leukemia by regulating the p53/p21/PCNA/eIF4E pathway. Besides from the tight correlation with the high relapse-free survival of breast cancer patients, overexpression of SerRS also suppressed the growth of cervical tumor xenografts by inducing cellular senescence *in vivo*, indicating that SerRS might have a certain degree of antitumor activity (Li Y. et al., 2019). Mechanistically, SerRS interacted with the telomere DNA repeats and recruited more protection of telomeres 1 (POT1) proteins to telomeres in the nucleus, thereby blocking telomerase recruitment and telomere elongation (Li Y. et al., 2019). These results indicate that SerRS may inhibit tumorigenesis by inducing cellular senescence. Mo et al. (2016) discovered that glycyl-tRNA synthetase (GRS) had the strongest correlation with mortality by analyzing the expression of 20 ARSs in 3557 breast cancer patients. Neddylation is a post-translational modification that attaches ubiquitin-like protein NEDD8 to its substrates and is implicated in cancer progression (Heo et al., 2020; Zhou X. et al., 2020). In particular, GRS captured and protected NEDD8-conjugated Ubc12 (activated E2) by binding to the APPBP1 subunit of E1, thereby increasing the global level of neddylation (Mo et al., 2016). Indeed, knockdown of GRS decreased the levels of NEDD8-conjugated Ubc12 and cullin neddylation in HeLa cells, leading to cell cycle arrest (Mo et al., 2016).

## Potential Clinical Applications of ARSs in Cancer

### ARSs as Potential Biomarkers

Meaningfully, certain ARSs can be used as diagnostic and prognostic biomarkers for cancer patients (Table 1). The levels of NRS and ERO1-like protein alpha (ERO1L) were higher in lung adenocarcinoma tissues compared with neighboring normal tissues, and their levels were positively associated with lymph node metastasis (Hsu et al., 2016). At the same time, ERO1L overexpression was associated with the poor survival rate of patients with early adenocarcinoma. Importantly, NARS or ERO1L knockdown could inhibit the growth and migration of adenocarcinoma cells (Hsu et al., 2016). These findings indicate that NRS and ERO1L not only play a role in cancer progression, but may also serve as novel markers for lung adenocarcinoma. Kim E.Y. et al. (2017) found that MRS was frequently overexpressed in cancer tissues from LSL-Kras G12D and LSL-Kras G12D:p53^fl/fl^ mice and human lung cancer tissues. Importantly, MRS could regulate the activity of mTORC1 signaling, and its overexpression was associated with poor prognosis in NSCLC, suggesting that MRS might contribute to the development of lung cancer (Kim E.Y. et al., 2017). Furthermore, the levels of nuclear KRS were significantly predictive of disease-free survival in NSCLC patients (Boulos et al., 2017).

**TABLE 1:** Aminoacyl-tRNA synthetases (ARSs) as potential biomarkers in cancer.

ARSs	Cancer type	Expression status	Effects	References
AIMP2-DX2	Lung cancer	Upregulation	The ratio of AIMP2-DX2 to AIMP2 was negatively correlated with patient survival.	Choi et al., 2011
EPRS	Breast cancer	Upregulation	Related to poor clinical outcomes in breast cancer.	Qi et al., 2019
SerRS	Breast cancer	Downregulation	High levels of SerRS were tightly correlated with the high relapse-free survival.	Li Y. et al., 2019
NRS	Lung cancer	Upregulation	Positively associated with lymph node metastasis.	Hsu et al., 2016
MRS	Lung cancer	Upregulation	Associated with poor prognosis in NSCLC.	Kim E.Y. et al., 2017
KRS	Lung cancer	Upregulation	Predicted disease-free survival in NSCLC patients.	Boulos et al., 2017
KRS	Colon cancer	Upregulation	Had a good diagnostic value for colorectal cancer.	Suh et al., 2020
TRS	Ovarian cancer	Upregulation	Positively correlated with disease stage and VEGF levels.	Wellman et al., 2014
AIMP3	Bladder cancer	Downregulation	Predicted the overall survival following radiotherapy and disease recurrence in MIBC patients.	Gurung et al., 2015

Additionally, the plasma KRS levels of patients with colorectal cancer were higher than those of healthy controls, and the levels were significantly reduced after surgery (Suh et al., 2020). Meanwhile, KRS levels were highly correlated with the number of polyps in the colorectal cancer mouse model. Notably, KRS had a good diagnostic value for colorectal cancer, and its diagnostic capability and specificity were better than those of current biomarkers, including carcinoembryonic antigen (CEA) and carbohydrate antigen 19-9 (CA19-9) (Suh et al., 2020). TRS levels in human ovarian cancer tissues were positively correlated with disease stage and vascular endothelial growth factor (VEGF) levels (Wellman et al., 2014). TRS could be secreted by cancer cells as a cellular stress response (Wellman et al., 2014). The serum TRS levels of patients were associated with tumor TRS and VEGF, but not with disease stage. These data indicate that TRS may be involved in tumor angiogenesis and serve as a potential biomarker for the diagnosis of ovarian cancer. Intriguingly, AIMP3 expression was reduced in muscle-invasive bladder cancer (MIBC) and was associated with genomic instability and radiation resistance (Gurung et al., 2015). AIMP3 levels could predict the overall survival following radiotherapy and disease recurrence in MIBC patients, which provided new ideas for the radiotherapy or chemo-radiotherapy of bladder cancer (Gurung et al., 2015).

### ARSs and Therapeutic Applications

**FIGURE 4:**
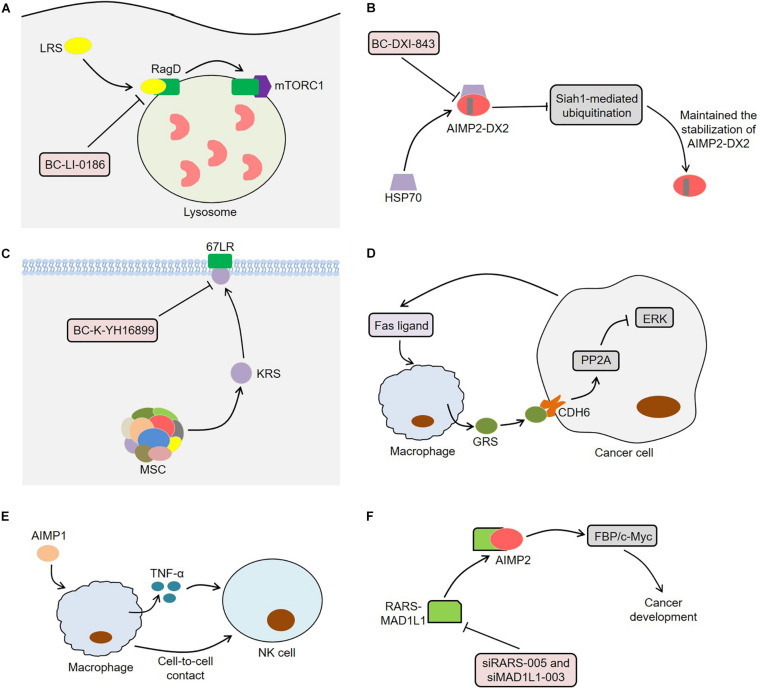
Aminoacyl-tRNA synthetases and potential therapeutic applications. **(A)** BC-LI-0186 binds to the RagD interacting site of LRS, thus suppressing the lysosomal localization and activity of mTORC1. **(B)** BC-DXI-843 promotes the degradation of AIMP2-DX2 by blocking the interaction between AIMP2-DX2 and HSP70, thereby inducing cancer cell apoptosis. **(C)** BC-K-YH16899 not only blocks the interaction of KRS and 67LR by binding to KRS, but also reduces the KRS membrane localization by restraining the flexible N-ext of KRS. **(D)** Macrophages secretes GRS under the stimulation of Fas ligand released by tumor cells. The secreted GRS interacts with specific ERK-activated tumor cells through CDH6, thereby enhancing PP2A activity by releasing PP2A from CDH6. The activated PP2A inhibits ERK signaling by binding to ERK and thus induces apoptosis of cancer cells. **(E)** AIMP1 inhibits the lung metastasis of melanoma cells in mice through macrophage-mediated activation of NK cells. AIMP1 induces macrophages to produce TNF-α, which partially activates NK cells. Meanwhile, the activation of NK cells requires the direct contact between NK cells and macrophages. **(F)** The knockdown of RARS-MAD1L1 using siRARS-005 or siMAD1L1-003 inhibits cancer development by suppressing FBP/c-Myc pathway. ARSs, aminoacyl-tRNA synthetases; LRS, leucyl-tRNA synthetase; mTORC1, mammalian target of rapamycin complex 1; AIMP2-DX2, AIMP2 lacking exon 2; HSP70, heat shock protein 70; KRS, lysyl-tRNA synthetase; 67LR, 67-kDa laminin receptor; GRS, glycyl-tRNA synthetase; CDH6, cadherin-6; PP2A, phosphatase 2A; ERK, extracellular signal-regulated kinase; AIMP1, ARS-interacting multifunctional protein 1; TNF-α, tumor necrosis factor α; NK cell, natural killer cell; RARS, arginyl-tRNA synthetase; MAD1L1, mitotic arrest deficient 1-like 1; FBP, FUSE-binding protein.

Since ARSs are closely related to tumor development, they may serve as potential targets for cancer treatment (Figure 4). LRS was often overexpressed in lung cancer tissues and performed an important function in the activation of mTORC1 and cell growth (Kim et al., 2019). The novel LRS inhibitor BC-LI-0186 could inhibit mTORC1 signaling, resulting in cytotoxicity to NSCLC cells and anticancer effects in K-ras mouse lung cancer model (Kim et al., 2019). In this process, BC-LI-0186 bound to the RagD interacting site of LRS, thus suppressing the lysosomal localization and activity of mTORC1 (Kim J.H. et al., 2017). Beltran et al. (2014) observed that the engineering interference peptides targeting Engrailed 1 (EN1) induced apoptosis of cancer cells and increased the sensitivity of resistant breast cancer cells to chemotherapy drugs by blocking the function of EN1. Interestingly, these interference peptides could also regulate downstream EPRS effectors by binding to EPRS in SUM149PT cells, indicating that EPRS inhibition might act as a potential treatment for breast cancer. Furthermore, BC-DXI-843, a sulfonamide based AIMP2-DX2 inhibitor, promoted the degradation of AIMP2-DX2 by blocking the interaction between AIMP2-DX2 and HSP70, thereby inducing cancer cell apoptosis (Sivaraman et al., 2020). Of note, BC-DXI-843 effectively reduced the tumorigenicity of H460 cells in mice (Sivaraman et al., 2020). Similarly, aminophenylpyrimidine 3 could exert anti-tumor effects by inhibiting AIMP2-DX2 in H460 and A549 cells (Lee et al., 2020).

Compound BC-K-YH16899 inhibited KRS-mediated cancer metastasis *in vivo* without affecting the catalytic activity of KRS (Kim D.G. et al., 2014). Specifically, BC-K-YH16899 not only blocked the interaction of KRS and 67LR by binding to KRS, but also reduced the KRS membrane localization by restraining the flexible N-ext of KRS. These findings indicate the potential of KRS as a target for antimetastatic therapy (Kim D.G. et al., 2014). In fact, the molecular structure of some ARSs contains a zinc atom, a sulfonamide binding scaffold, which provides an idea for the development of ARS inhibitors (Sankaranarayanan et al., 2000; Bilokapic et al., 2006; Kumar et al., 2015). Strikingly, macrophages secreted GRS under the stimulation of Fas ligand released by tumor cells (Park et al., 2012). The secreted GRS interacted with specific ERK-activated tumor cells through cadherin-6 (CDH6), thereby enhancing phosphatase 2A (PP2A) activity by releasing PP2A from CDH6 and reducing PP2A phosphorylation (Park et al., 2012). Next, the activated PP2A inhibited ERK signaling by binding to ERK and reducing its phosphorylation, thus inducing apoptosis of cancer cells. Meaningfully, GRS significantly suppressed tumor growth in a xenograft model using HCT116 cells, indicating that GRS might be a potential target for cancer treatment (Park et al., 2012). Furthermore, AIMP1 could inhibit the lung metastasis of melanoma cells in mice through macrophage-mediated activation of natural killer (NK) cells (Kim M.S. et al., 2017). On the one hand, AIMP1 significantly induced macrophages to produce TNF-α, which partially activated NK cells (Kim M.S. et al., 2017). On the other hand, the activation of NK cells induced by AIMP1 required the direct contact between NK cells and macrophages. The fusion protein RARS-mitotic arrest deficient 1-like 1 (MAD1L1) activated the FBP/c-Myc pathway by interacting with AIMP2, thereby inducing cancer stem cell-like phenotypes (Zhong et al., 2018). The knockdown of RARS-MAD1L1 reduced the growth and colony formation of cancer cells *in vitro*, indicating that RARS-MAD1L1 inhibition might be a new strategy for cancer treatment (Zhong et al., 2018).

## Conclusion and Future Perspective

Traditionally, ARSs are considered as housekeeping molecules that mainly participate in protein synthesis by catalyzing the aminoacylation of tRNAs. Therefore, these enzymes are necessary for maintaining cell homeostasis and normal physiological functions of the body. In fact, in addition to this main function, ARSs also play an important role in various pathological processes (Stephen et al., 2018; D’Hulst et al., 2020; Wu et al., 2020). Strikingly, ARSs are closely associated with tumor biology. On the one hand, ARSs are involved in the progression of various cancers, such as colon cancer, lung cancer, breast cancer, gastric cancer and pancreatic cancer. Among them, some ARSs such as IARS2, KRS and NRS have been reported to promote the development of cancer (Kim et al., 2012; Fang et al., 2018; Yeom et al., 2020), while AIMPs, SerRS and GRS usually exert anti-tumor effects (Park et al., 2012; Li Y. et al., 2019; Zhou Z. et al., 2020). On the other hand, ARSs can be used not only as biomarkers for cancer diagnosis and prognosis, but also as potential targets for inhibiting cancer growth and metastasis.

Aminoacyl-tRNA synthetases have evolutionarily conserved enzymatic mechanisms, and the understanding of their molecular structure is conducive to the rational design of drugs. Indeed, the therapeutic applications of ARSs have begun to be explored in human diseases, especially in infections, cancer and autoimmune diseases (Keller et al., 2012; Baragana et al., 2019; Kwon et al., 2019). Since ARSs are directly involved in protein synthesis, we should pay more attention to the possible cytotoxicity when studying the potential applications. As described in the article, ARSs and AIMPs promote or inhibit cancer development through different molecular mechanisms. Therefore, a variety of treatment modalities targeting ARSs can be explored, such as compound inhibitors, peptides and gene therapy. Considering that amino acids are the substrates of ARSs, the involvement of high-protein diets or ketogenic diets in the development of tumors through regulating ARSs may be an interesting research direction. Moreover, some types of cancer are described to depend on specific amino acids (Tang et al., 2017; Salzer et al., 2018), and certain ARSs can regulate amino acid transport (Moore et al., 1977; Miyanokoshi et al., 2018). Therefore, further studies are needed to determine whether ARSs influence cancer development by regulating amino acid transporters. All in all, we summarized the roles of ARSs in tumorigenesis, with the purpose of providing new ideas for cancer treatment.

## Author Contributions

ZZ and BS contributed toward the concept and manuscript writing. AN participated in the literature search and discussion. DY and MB revised and supervised overall project. All authors read and approved the final version of manuscript.

## Conflict of Interest

The authors declare that the research was conducted in the absence of any commercial or financial relationships that could be construed as a potential conflict of interest.
